# Generation of Endogenous Promoter-Driven Luciferase Reporter System Using CRISPR/Cas9 for Investigating Transcriptional Regulation of the Core Clock Gene *BMAL1*

**DOI:** 10.3390/biomedicines10123108

**Published:** 2022-12-01

**Authors:** Chengxi Sun, Chen Li, Wen Liu, Helgi B. Schiöth

**Affiliations:** 1 Department of Surgical Sciences, Uppsala University, 751 24 Uppsala, Sweden; 2 Department of Clinical Laboratory, Qilu Hospital, Cheeloo College of Medicine, Shandong University, Jinan 250012, China; 3 Department of Medical Biochemistry and Microbiology, Uppsala University, 752 36 Uppsala, Sweden

**Keywords:** luciferase, circadian rhythm, CRISPR/Cas9, endogenous transcriptional regulation, *BMAL1*

## Abstract

Humans and other organisms are continuously exposed to thousands of chemicals through the atmosphere, drinking water, food, or direct contact. A large proportion of such chemicals are present in very low concentrations and may have synergistic effects, even at their no-observed-adverse-effect level (NOAEL). Complex mixtures of contaminants are very difficult to assess by traditional toxicological methods. There is increasing attention on how different pollutants induce adverse physiological functions in the human body through effects on the circadian rhythm. However, it is very difficult to screen for compounds with circadian-rhythm-disrupting effects from a large number of chemicals or their complex mixtures. We established a stable firefly luciferase reporter gene knock-in U2-OS cell line by CRISPR/Cas9 to screen circadian-rhythm-disrupting pollutants. The luciferase gene was inserted downstream of the core clock gene *BMAL1* and controlled by an endogenous promoter. Compared to detection systems using exogenous promoters, these cells enable the detection of compounds that interfere with the circadian rhythm system mediated by *BMAL1* gene expression. The U2-OS knock-in cells showed BMAL1 and luciferase activity had parallel changes when treated with BMAL1 inhibitor and activator. Furthermore, the luciferase reporter gene has high sensitivity and is faster and more cost-effective than classic toxicology methods. The knock-in cell line can be used for high-throughput and efficient screening of circadian-rhythm-disrupting chemicals such as drugs and pollutants.

## 1. Introduction

Humans are exposed to many chemicals, and pollution is the main cause of environment-related disease and premature death in the world, especially in low-income to middle-income countries [1,2]. Humans ingest various pollutants through pesticides, plastic products, food, building materials, kitchen utensils, pharmaceuticals, and personal care products [3]. Pollutants have been associated with human metabolic diseases, reproductive dysfunction, immune system diseases, and cancers by epidemiological data and laboratory studies [4,5,6,7]. The deleterious effects of several pollutants have been demonstrated in insect, amphibian, bird, and mammalian models [8,9,10,11,12]. Moreover, humans are exposed to pollutants for much longer than laboratory exposure times, and the types of pollutants exposed are far more complex than those in experimental models [4,12]. Pollutants are usually present in very low concentrations in the environment, and many are at their no-observed-adverse-effect level. Mixtures of these contaminants may synergistically exert biological effects and no longer conform to the stoichiometric response curve [10,11,13]. The biological effects of chemicals or mixtures are difficult to assess using traditional toxicological methods [14,15,16]. We lack validated models and methods to study the damage from exposure to high numbers of chemicals and their mixtures in humans.

Epidemiological studies are currently the main method to assess the impact of pollutants on human health [17]. However, laboratory data and efficient laboratory research models for the rapid screening of pollutant mixtures are insufficient. In traditional toxicology studies, drug concentrations are often set far greater than the concentrations of pollutants in the environment. For example, for PFOA and PFOS, the detectable functional changing treatment concentration has often been set at 10–100 μM, while the concentration in the environment and human blood is about 10–20 nM [18]. However, the experimental data obtained at this concentration cannot fully reflect the real biological effects in the environment. If the treatment concentration is reduced to around 10 nM, there is a high probability that the results are not transferrable to the natural situation. The role of a single pollutant is usually studied in experimental models, and the presence of pollutants in the environment acts as a mixture that may have synergistic and antagonistic actions [13,14]. If the biological effects of mixtures are studied in experimental studies, a huge number of combinations need to be tested, which is difficult to achieve with existing methods [15,17]. Therefore, high-sensitivity and high-throughput methods need to be established to study the biological effects of pollutants and their mixtures.

Many environmental pollutants can mimic natural hormones, leading to the disturbance of hormone homeostasis, which has been confirmed by a large number of wild animal studies [10,12]. Recent studies suggest that some of these pollutants can cause circadian rhythm disturbances [12,19]. The circadian clock is an evolutionarily conserved 24 h oscillator rhythm driven by endogenous clock genes to anticipate and adapt to the external environment in eukaryotic life, from cyanobacteria to mammals [20,21,22,23]. The biochemical, physiological, and behavioral functions of organisms are controlled by a group of autoregulatory transcription–translation genes [24,25,26]. In mammals, the circadian pacemaker is composed of interlocked feedback loops with the core transcription factors *CLOCK* and *BMAL1*, which drive the transcription of the Period (*PER1*, *PER2*) and Cryptochrome (*CRY1*, *CRY2*) genes [23]. The CLOCK/BMAL1 heterodimer can interact with many transcription factors directly, which regulate the cell cycle, cell proliferation, and cell metabolism, and also plays an important role in cancer [27,28]. Microarray data from the liver and heart tissue have indicated that up to 10% of all the genes that are transcribed show robust rhythm during the circadian cycle [29]. Circadian rhythm disruption seems to be implicated in several pathologic conditions, including tumorigenesis and the progression of cancer [30,31]. The circadian rhythm affects the progression and chemoresistance of a variety of tumors, including breast cancer, pancreatic cancer, skin cancer, leukemia, and colon cancers, by DNA damage repair and interacting with oncogenes or tumor suppressors [32,33,34,35,36,37,38]. BMAL1, as the core member of the circadian system, regulates tumor cell apoptosis, cell-cycle progression and DNA damage response, and homoeostasis.

In this study, we developed a luciferase reporter system of the U2-OS cell line for investigating the *BMAL1* mRNA expression level driven by endogenous transcriptional regulation using CRISPR/Cas9 technology. The stable luciferase reporter U2-OS cell line could be an efficient tool for screening circadian-rhythm-disrupting chemicals.

## 2. Methods and Material

### 2.1. Design of sgRNA Targeting BMAL1 Locus

The analysis of the C-terminal coding region of the *BMAL1* gene (ENST00000389707.8) was undertaken by UCSC (https://genome.ucsc.edu, accessed on 3 November 2021). It provided a number of potential Cas9 guide sites in exon 20 of *BMAL1*. An sgRNA across the stop codon of *BMAL1* was chosen and synthesized by Integrated DNA Technologies (IDT). The sequence of the sgRNA was 5′-GCAACATGTAGTGTTTACAG-3′.

### 2.2. Construction of Donor Plasmids

Homologous arms were synthesized to oligo DNAs as a PCR template. Two pairs of primers were used to amplify the left and right homologous arms (Table 1). A P2A sequence was inserted between the left homologous arm and the luciferase gene for the self-cleaving of the peptide after translation. A CMV-luciferase-EGFP-Cre-SV40 promoter-puromycin expression cassette was amplified from pF CAG luc-EGFP-Cre puro plasmid (Addgene, #67503) by PCR using the following conditions: DNA denaturation at 98 °C for 3 min, followed by 32 cycles of denaturation at 98 °C for 10 s, annealing at 62 °C for 20 s, extension at 72 °C for 2 min, and terminal extension at 72 °C for 5 min (PF-luc-puro, PR-luc-puro). The PCR products of the homologous arms and expression cassette were mixed at a 1:1:1 mole ratio as templates, and amplification was performed at 98 °C, 3 min for denaturation, followed by 32 cycles of denaturation at 98 °C for 10 s, annealing at 62 °C for 20 s, extension at 72 °C for 2 min 30 s, terminal extension at 72 °C for 5 min to obtain the Left arm-Luc expression cassette-Right arm fragment. All of the PCR products were purified by gel extraction (Qiagen). The primers for amplifying the upstream and downstream homologous arms are listed in Table 1.

The pcDNA3.1+ plasmid was digested with *PstI* and *KpnI*. The liner pcDNA3.1+ plasmid and the Left arm-Luc expression cassette-Right arm DNA fragments were mixed at a 1:5 mole ratio and incubated with T4 DNA ligase at 22 °C for 1 h. Then, the ligase product was co-transformed into competent *E. coli DH5 α*. The plasmids were named pcDNA3.1-Left-Luc-RIGHT and extracted for sequencing to validate the recombination.

### 2.3. Cell Culture

Human bone osteosarcoma epithelial cells U2-OS were cultured in DMEM with 10% (*v*/*v*) heat-inactivated fetal bovine serum and 50 μg/mL streptomycin and penicillin (Thermo Fisher Scientific, Waltham, MA, USA). The cell culture was maintained in a humidified 5% CO_2_ incubator at 37 °C.

### 2.4. Nucleo Transfection

Transfection was performed by nucleofection (Amaxa^TM^ Cell Line Nucleofector^TM^ Kit). The U2-OS cells were seeded in a T75 flask 2 days before nucleofection to reach 70% confluence by the day of transfection. After trypsinization, 2 × 10^5^ cells were resuspended in 70 µL of nucleofector solution. To perform the CRISPR/Cas9-based gene-editing, Cas9 protein was ordered from SYNTHEGO. To prepare the sgRNA-Cas9 protein complex, 6 µL of sgRNA (30 pmol/μL), 1 µg of liner pcDNA3.1-Left-Luc-RIGHT plasmid, and 1 µL of Cas9 (20 pmol/μL) were added to 23 µL of Nucleofector solution. A volume of 70 µL of cell suspension was added to 30 µL of pre-complexed Cas9 protein and sgRNA for a total transfection volume of 100 µL. All of the 100 µL cell-sgRNA-Cas9 solution was transferred into an aluminum cuvette and electroporated using the T-024 program with a nucleofector-2 machine. A volume of 500 µL of prewarmed complete medium was added to the cuvette immediately. The cell suspension was transferred into a 1.5 mL Eppendorf tube and kept at 37 °C for 30 min for recovery. Then, 300 µL of cell suspension was added to each 24-well plate, and the medium was changed 24 h post-nucleofection.

### 2.5. Establishment of Stable Cell Line

At 48 h post-nucleofection, U2-OS bulk cells were selected with puromycin (1 μg/mL) and maintained until the cultures were recovered (~1 week). The cells were resuspended to a concentration of 10 cells/mL by serial dilution and seeded into a 96-well plate with 100 μL/well. The cells were cultured in the 96-well plate with puromycin selection (1 μg/mL) for 1 week and screened by EGFP using a fluorescence microscope (Nikon 90i, Tokyo, Japan). The EGFP-positive clones were expanded in a 6-well plate with puromycin (1 μg/mL).

### 2.6. Cell Viability and Cell Morphology

PrestoBlue was used to measure cell viability (Thermo Fisher Scientific). A total of 5000 U2-OS or U2-OS-Luc cells were seeded in a 96-well plate and cultured for 24 h. A volume of 10 μL/well PrestoBlue was added, and the culture was incubated at 37 °C for 2 h. Absorbance at 562 nm was detected using a plate reader (Thermo Fisher Scientific, Waltham, MA, USA). The images of cell morphology for U2-OS and U2-OS-Luc cells were taken using an optical microscope (Nikon, Tokyo, Japan).

### 2.7. PCR-Based Detection

The genomic DNA of U2-OS-Luc cells was extracted (Qiagen) for integrated validation by PCR. A pair of primers was designed for the luciferase gene to detect the knock-in in the U2-OS cell line (Table 1). DNA with Cas9-mediated integration of the donor template generates a band of 1650 bp. Approximately 5 × 10^4^ U2-OS-Luc cells were seeded in a 24-well plate and treated with SR9009 and SR8278 (Thermo Fisher Scientific) for 24 h. Then, the RNA was extracted using the PureLink RNA Kit (Thermo Fisher Scientific). The extracted RNA was reverse transcribed using a TaqMan miRNA reverse transcription kit (Thermo Fisher Scientific). The quantification of *BMAL1* mRNA by TaqMan Real-Time PCR was carried out according to the manufacturer’s instructions. The target gene *BMAL1* expression level was normalized based on the values of RPL13A RNA expression (Integrated DNA Technologies IDT, Coralville, LA, USA).

### 2.8. Immunoblot Analysis

U2-OS and U2-OS-Luc cells were washed with PBS and then lysed using RIPA buffer (Cell Signaling, Stockholm, Sweden) with a protease inhibitor cocktail (Roche, Stockholm, Sweden). The collected cell lysate was used to determine the protein concentration with a BCA Kit (Thermo Fisher Scientific). For each sample, 20 µg of protein was resolved in a 4–15% SDS-PAGE gel (Bio-Rad, Solna, Sweden) along with a protein ladder (Bio-Rad, Solna, Sweden) and transferred onto PVDF membranes. The transferred membranes were blocked in 5% milk in TBST (25 mM Tris-HCl, pH 7.4, 125 mM NaCl, 0.05% Tween 20) for 1 h and incubated with the primary antibodies at 4 °C overnight. After the membrane was washed thrice with TBST for 7 min, it was incubated with horseradish–peroxidase-coupled isotype-specific secondary antibodies (Dako) for 1 h at room temperature. Detection was performed by SuperSignal™ West Atto Ultimate Sensitivity Substrate according to the manufacturer’s instructions (Thermo Fisher Scientific). Primary antibodies for BMAL1 and *p-*BMAL1 were from Thermo Fisher Scientific.

### 2.9. Luciferase Assay

A total of 5000 cells was seeded into a 96-well plate 24 h before luciferase assay. The medium was removed, and the cells were washed with PBS. Lysis buffer was added to the plate (20 μL/well). Then, 100 μL of D-Luciferin (10 μM) was added to each well, and luminescence measurements were performed using a luminometer (EnSpire™ Multimode Plate Reader, PerkinElmer, Waltham, MA, USA).

### 2.10. Statistical Analysis

The data were analyzed using GraphPad Prism 5.0 software (GraphPad, San Diego, CA, USA). The differences between experimental groups were tested for significance using either Student’s *t*-test or one-way analysis of variance (ANOVA). The *p*-values < 0.05 were considered to be statistically significant.

## 3. Results

### 3.1. Construction of Donor Template

**Figure 1:**
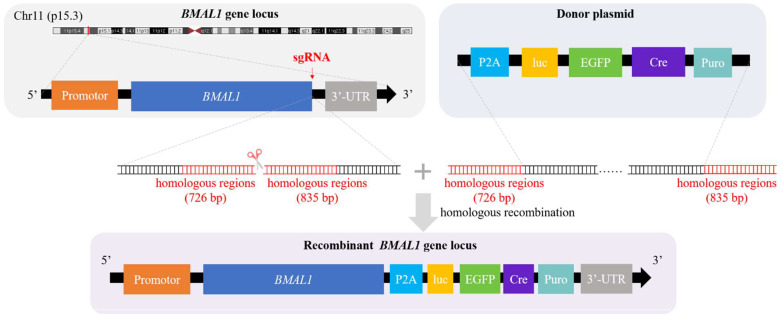
Schematic model of establishing the U2-OS-Luc cell line targeting the *BMAL1* gene locus. Cas9-mediated DNA double-strand break was performed by Cas9 protein and *BMAL1* targeting sgRNA. A P2A-luciferase-EGFP-Cre-SV40-puromycin expression cassette was inserted into the terminal code of *BMAL1* by homologous recombination. Luc: luciferase.

**Figure 2:**
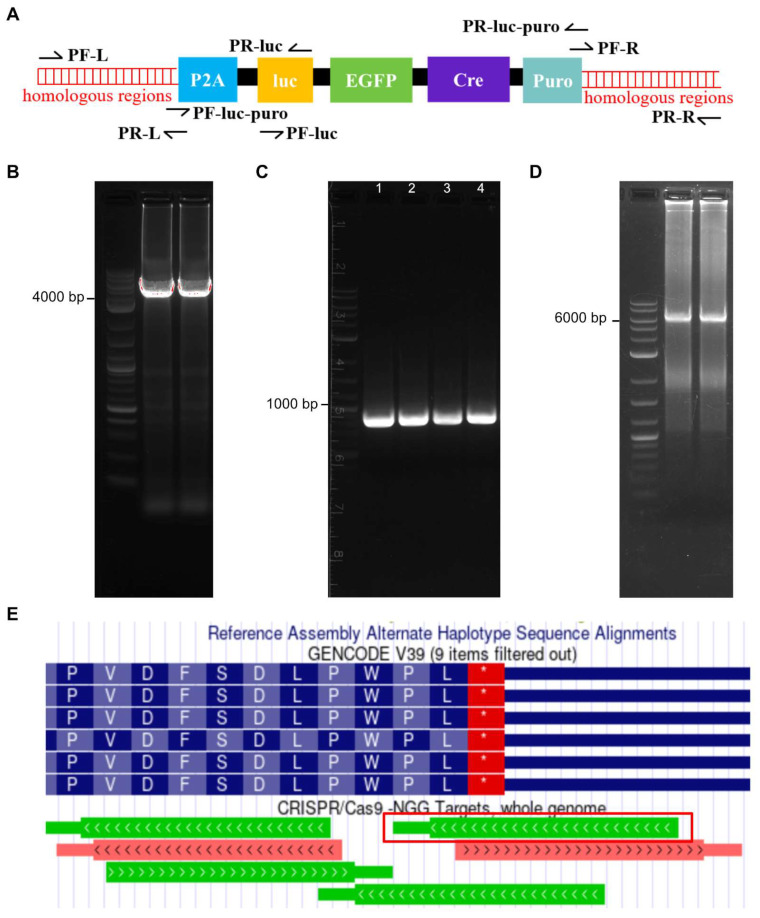
The strategy to construct luciferase homologous donor vector. (**A**) The primer location and distance for constructing the donor plasmid. The sequence and purpose of primers are shown in Table 1. (**B**) Electrophoresis band of the donor plasmid (P2A-luciferase-EGFP-Cre-SV40-puromycin cassette with homologous arms to *BMAL1* gene locus) PCR product. (**C**) The left homologous arm (lane 1–2, 726 bp) and right homologous arm (lane 3–4, 835 bp) are located upstream and downstream of the stop codon site of the *BMAL1* gene, respectively. (**D**) Electrophoresis band for the PCR product of Left-Luc-Right fragment. (**E**) The sgRNAs of *BMAL1* stop code site in the genome (https://genome.ucsc.edu, accessed on 3 November 2021). The sgRNA in red frame was used to establish the stable U2-OS cell line. The sequence of the sgRNA is 5′-GCAACATGTAGTGTTTA↓CAG-3′, and the arrow is the Cas9 cleavage site.

In order to generate the *BMAL1* endogenous-promoter-driven luciferase reporter U2-OS cell line, the terminal code of *BMAL1* was removed and replaced by a P2A-luciferase-EGFP-CRE-SV40-puromycin expression cassette (Figure 1). P2A self-cleaving peptide can induce ribosomal skipping during translation to generate polyproteins from one transcript. The C-terminal coding region of the *BMAL1* gene was analyzed by UCSC (Figure 2E). The guide RNA on chr11:13386748-13386770 across the terminal code of *BMAL1*, with an MIT Guide Specificity Score of 78, was used to mediate the gene targeting.

To construct the donor vector, the P2A-luciferase-EGFP-Cre-SV40-puromycin expression cassette fragment was amplified from the pF CAG Luc-EGFP-Cre puro plasmid, which includes the luciferase gene, EGFP, and Cre fusion protein and a puromycin select marker (Figure 2A). The Left (786 bp) and Right (835 bp) homologous arms were designed upstream and downstream of the terminal codon site of *BMAL1*, respectively (Supplementary Materials S1–S3). The P2A-luciferase-EGFP-Cre-SV40-puromycin expression cassette was amplified by PCR using pF CAG Luc-EGFP-Cre puro plasmid as a template (Figure 2B). The homologous arms were synthesized as DNA oligos, and PCR was performed to obtain the double-stranded DNA (Figure 2C). The primers of the homologous arms and expression cassette were designed with 20–25 bp overlapping ends for fusion PCR to combine the three DNA fragments (Table 1). The PCR products of homologous arms and P2A-luciferase-EGFP-Cre-SV40-puromycin expression cassette were combined to a Left-Luc-Right fragment (Figure 2D). The primer of PF-LEFT and PR-RIGHT have homologous sequences at the *PstI* and *KpnI* restriction enzyme sites of pcDNA3.1+, respectively. The pcDNA3.1+ plasmids and Left-Luc-Right were digested with *PstI* and *KpnI* and joined by T4 ligase. After transformation, the plasmid was sequenced to validate the recombination.

### 3.2. Generation of the U2-OS-Luc Cell Line

**Figure 3:**
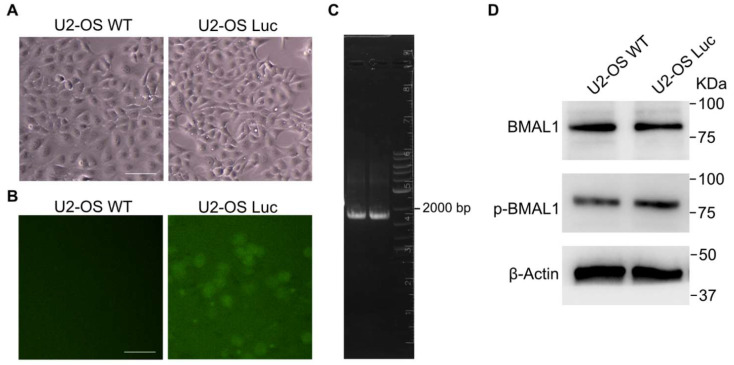
Cell biological characteristics of the CMV-luciferase-EGFP-Cre-puromycin expression cassette knock-in U2-OS cell line. (**A**) Cell morphology of wild-type U2-OS and U2-OS-Luc cell lines. Scale bar = 50 μm. (**B**) The U2-OS-Luc cells can express EGFP. Scale bar = 50 μm. (**C**) Genomic DNA was extracted from U2-OS-Luc cells, and PCR was performed to confirm the luciferase gene insert (Lanes 1 and 2). The primers’ location and distance are shown in Figure 2A. (**D**) BMAL1 and *p*-BMAL1 expression levels in wild-type U2-OS and U2-OS-Luc cell lines were detected using Western blot.

We performed a nucleo transfection using a co-transfect liner donor vector, Cas9 protein and sgRNA into a U2-OS cell. The modified cells were selected with puromycin for 1 week. Then, single clones were isolated using a limited dilution assay and cultured for 10 days. The cell morphology of the wild-type U2-OS and U2-OS-Luc cell lines is identical (Figure 3A). The single clones that can express EGFP were chosen for further validation (Figure 3B).

### 3.3. Validation of the U2-OS-Luc Cell Line

**Figure 4:**
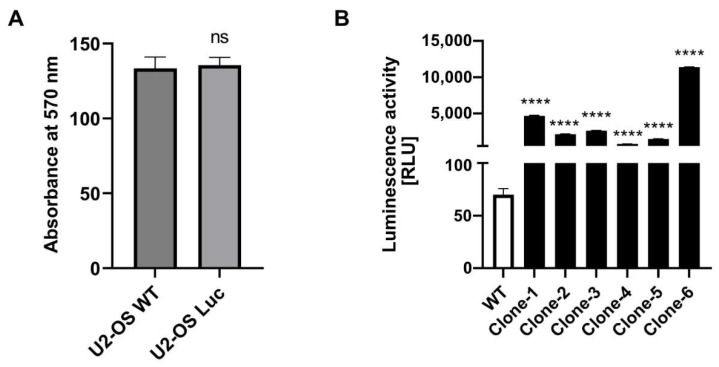
The phenotype of U2-OS stable cell line. (**A**) Cell viability was detected in wild-type U2-OS and U2-OS-Luc cell lines with six replicates by Prestoblue Assay (*n* = 3). ns., not significant, Student′s *t*-test. Data are presented as mean ± SEM. (**B**) The luciferase activity of isolated U2-OS-Luc single clones. **** *p* < 0.0001 compared to WT U2-OS cell, Student’s *t*-test.

Genomic DNA was extracted from U2-OS-Luc cells, and PCR was performed to confirm the luciferase gene insert (Figure 3C). The cell morphology of the wild-type and CRISPR/Cas9 knock-in U2-OS cells is consistent and unchanged. Immunoblot analysis demonstrated that BMAL1 and *p*-BMAL1 have an equivalent expression level in wild-type and luciferase knock-in U2-OS cell lines (Figure 3D). A luciferase assay was performed on 16 clones after puromycin selection and EGFP screening. Six of the 16 clones displayed luciferase activity. The wild-type and U2-OS-Luc cell lines have the same cell viability (Figure 4A). Clone 6, with the highest luciferase activity, was chosen for further validation (Figure 4B).

### 3.4. Luciferase Reporter Gene Responds to Circadian Rhythm in U2-OS Cell Line

**Figure 5:**
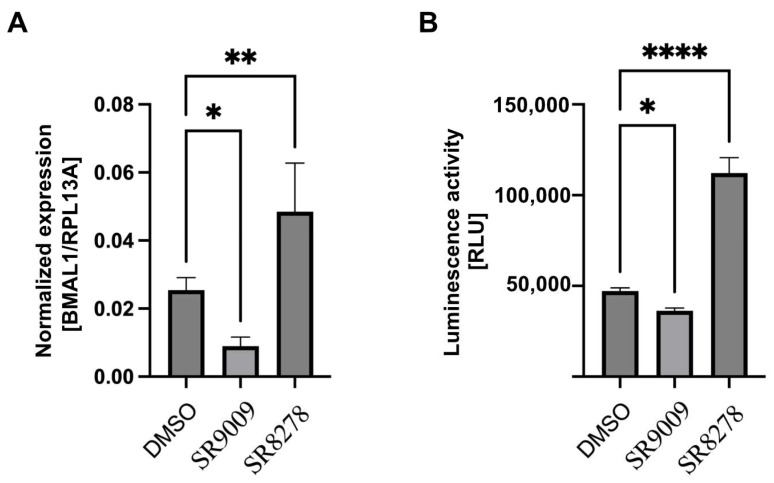
Validation of the *BMAL1* promoter–promoter-driven luciferase reporter system in U2-OS-Luc cell line. (**A**) Relative mRNA levels of *BMAL1* gene in U2-OS-Luc cells treated with BMAL1 activator SR9009 and inhibitor SR8278 (*n* = 3). * *p* < 0.05 and ** *p* < 0.01 compared to DMSO control, Student′s *t*-test. (**B**) The luciferase activity in U2-OS-Luc cells treated with BMAL1 activator and inhibitor (*n* = 3). * *p* < 0.05, ** *p* < 0.01 and **** *p* < 0.0001 compared to DMSO control, Student’s *t*-test.

U2-OS cell line has a 24 h oscillator rhythm driven by *CLOCK/BMAL1* genes. The wild-type and knock-in U2-OS-Luc cell lines have the same dynamic of BMAL1 expression in circadian cycles, which illustrates that the gene editing did not impact the natural circadian rhythm (Figure 3D). For further verification, the REV-ERB agonist II, SR9009 and REV-ERB antagonist SR8278 were used as BMAL1 inhibitors and activators to detect the parallel of the transcription regulation of *BMAL1* and luciferase gene, respectively. The expression level of BMAL1 detected by qPCR and luciferase activity was inhibited or promoted by SR9009 and SR8278 treatment (Figure 5). It implied that the transcription of *BMAL1* and luciferase is controlled by the *BMAL1* promoter, and the transcription regulation can be represented by the activity of luciferase.

## 4. Discussion

Here we describe an endogenous-promoter–promoter-driven luciferase reporter system cell line made using CRISPR/Cas9 gene-editing technology, enabling high-throughput and sensitive circadian chemical screening with luciferase. The entire experimental scheme can be implemented using common equipment in the laboratory, and no special equipment is required. In our model, the luciferase gene was inserted downstream of *BMAL1* by homologous recombination within the same ORF of *BMAL1* and regulated by the *BMAL1* promoter (Figure 1). The *BMAL1* endogenous-promoter-controlled luciferase reporter system will be used to study the transcriptional regulation of chemicals on circadian core genes. Compared with exogenous reporter systems, it can well reflect the real biological process in vivo.

To establish the luciferase reporter system regulated by the endogenous promoter, we designed sgRNA at the stop codon of the *BMAL1* gene (Figure 2E). CRISPR-Cas9 was used to create a DNA double-strand break at the site of the stop codon of the *BMAL1* gene. U2-OS osteosarcoma cells, a model commonly used in studies of the human circadian rhythm in vitro, were used to establish a reporter system to evaluate transcriptional regulation of clock genes by exogenous promoters of *CLOCK* or *BMAL1* [39,40,41]. The luciferase expression cassette was then inserted into the U2-OS genome by homologous recombination. In order to validate whether recombinant cells can express exogenous proteins with biological activity or not, we performed experiments including puromycin selection, EGFP screening, and luciferase activity driven by the *BMAL1* promoter. Single clones were obtained after puromycin and EGFP screening (Figure 3B,C). The cell morphology and cell viability assays showed that the biological characteristics of the stably transfected cell lines were not altered (Figure 3A and Figure 4A). The results of Western blot showed that the protein levels of BMAL1 and p-BMAL1 were not changed in wild-type and knock-in U2-OS cells (Figure 3D). These results indicated that the insertion of the luciferase expression cassette did not affect the normal transcription and expression of the *BMAL1* gene. Then, single clones with high luciferase expression activity were further screened by luciferase activity (Figure 4B). The stable luciferase reporter cell line can be established within 20 days.

Circadian rhythm is a self-perpetuating oscillation with a 24 h periodicity and is synchronized with behavior and physiology in most organisms [20,21,29]. Disruptions in biological rhythms result in numerous disorders in organismal homeostasis linked to diverse pathogenic conditions, including cancer, which is supported by epidemiological evidence and laboratory data [31,32,33,34]. Using a *Drosophila* model, it has been reported that environmental pollutants can cause cell over-proliferation, affect lipid metabolism, and disrupt circadian rhythms [42,43,44]. The molecular circadian machinery is composed of a set of clock genes. The heterodimeric transcription factor complex *CLOCK/BMAL1* is the core of the circadian rhythm system [23]. In general, qPCR and Western blot are usually used to analyze the transcription and expression level of circadian genes. These methods are time-consuming and vary highly in some cases. Lentiviral-mediated integration was also used to investigate target gene functions, but the random insertion might affect genome homeostasis [45]. However, random insertion in the genome by lentiviral transduction may produce unknown effects on cells’ biological properties. Compared with these classical methods, analyzing gene transcription regulation using an endogenous promoter is more precise in revealing the unique chromatin domain or binding site for enhancers, repressors, or cofactors.

CRISPR/Cas9 induces the specific-site knock-in with high cleavage precision and efficiency. Furthermore, the luciferase reporter system is more reliable and cost-effective and can detect more subtle changes than other molecular biology methods. The U2-OS-Luc knock-in cell line we established in this study showed a parallel dynamic of mRNA level of *BMAL1* and luciferase activity after treatment with BMAL1 antagonists or agonists in the circadian circle (Figure 4). It can be used to investigate BMAL1 functional roles in circadian-related diseases and high-throughput screening for environmental pollutants and their mixture, which can disrupt *BMAL1* transcriptional activity.

Previous studies have established several methods, such as qPCR, Western blot, or reporter gene expression, to investigate transcriptional regulation by exogenous promoters. Plasmid- or lentiviral-mediated integration was also used to construct the stable cell line for exogenous-promoter-driven reporter gene expression [46,47]. To investigate endogenous-promoter-controlled transcription, activator-like effector nucleases (TALENs) and zinc finger nucleases (ZFNs) have been developed for targeting gene editing by homologous recombination [48]. However, these methods have high off-target mutation tendencies or low feasibility [49]. In recent years, the CRISPR/Cas9 system has provided an efficient method for gene editing [50]. CRISPR/Cas9 enables insertion, deletion, transcriptional activation, or transcriptional repression at precise genetic loci with reduced impact on genome homeostasis [51]. With the optimization of the structure of the Cas9 protein, it will be more widely used in the field of gene editing [52,53].

This model can become very useful for various reasons. Screening and evaluating the circadian-rhythm-disrupting effects of pollutants is of great significance for formulating environmental protection strategies and protecting human health. There are thousands of environmental pollutants; humans are chronically exposed to these compounds and the toxicity of most of them is unknown or underestimated. There may be synergistic effects between some of these pollutants that will not be detected using single chemicals [54]. In laboratory studies, there will be a huge number of combinations that are difficult to evaluate using traditional methods. At the same time, the concentration of pollutants in the environment is usually very low. Due to the existence of no-observed-adverse-effect level (NOAEL) phenomena, it is difficult to observe biological changes with traditional toxicological methods [55,56]. Therefore, it is necessary to develop a high-throughput, high-sensitivity method to screen pollutants for circadian disruption.

## Figures and Tables

**Table 1:** Primer sequence for development of an endogenously driven luciferase reporter U2-OS cell line for core clock gene *BMAL1*.

Name	Primer Sequence (5′-3′)	Use
PF-luc-puro	AGATGTCGAAGAGAATCCTGGACCGATGGAAGACGCCAAAAACATAAAG	PCR for luciferase expression cassette
PR-luc-puro	CTGTTGCCAAAGCAACATGTAGTGTTCCACATAGCGTAAAAGGAGCAACATAG	PCR for luciferase expression cassette
PF-L	GCTGGCTAGCGTTTAAACTTAAGCTTTGGAGGTCCAAGTTTGTGCCTGGAA	Infusion for homology domain
PR-L	TCCAGGATTCTCTTCGACATCT	Infusion for homology domain
PF-R	CTATGTTGCTCCTTTTACGCTATGT	Infusion for homology domain
PR-R	GGACTAGTGGATCCGAGCTCGGTACCAGAGAGGTGCAGCATTAGAGAAGCC	Infusion for homology domain
PF-luc	CCGGCGCCATTCTATCCTC	PCR validation of luciferase knock-in
PR-luc	CCTTTCGGTACTTCGTCCACA	PCR validation of luciferase knock-in
PF-*BMAL1*	TTAAGAGGTGCCACCAATCC	qPCR for *BMAL1*
PR-*BMAL1*	CTTCCCTCGGTCACATCCTA	qPCR for *BMAL1*

## Data Availability

The sgRNA was designed by by UCSC (https://genome.ucsc.edu). The pF CAG luc-EGFP-Cre puro plasmid was obtained from Addgene. The homologous arms sequence and donor DNA sequence are available in supplementary Material.

## Supplementary Materials

The following supporting information can be downloaded at: https://www.mdpi.com/article/10.3390/biomedicines10123108/s1, S1: Left homologous arm sequence of *BMAL1*. S2: Right homologous arm sequence of *BMAL1*. S3: The DNA sequence of P2A-EGFP-CRE-Puro cassette from Addgene (#67503).

## References

[1] Landrigan P.J., Fuller R., Acosta N.J.R., Adeyi O., Arnold R., Basu N.N., Baldé A.B., Bertollini R., Bose-O’Reilly S., Boufford J.I. (2018). The Lancet Commission on pollution and health. Lancet.

[2] Wasi S., Shams T., Masood A. (2013). Toxicological effects of major environmental pollutants: An overview. Environ. Monit. Assess..

[3] Ng A., Weerakoon D., Lim E., Padhye L.P. (2019). Fate of environmental pollutants. Water Environ. Res..

[4] Le Magueresse-Battistoni B., Hubert V., Danielle N. (2018). Environmental pollutants and metabolic disorders: The multi-exposure scenario of life. Front. Endocrinol..

[5] Joffe M. (2003). Infertility and environmental pollutants. Br. Med. Bull..

[6] Suzuki T., Hidaka T., Kumagai Y., Yamamoto M. (2020). Environmental pollutants and the immune response. Nat. Immunol..

[7] Boffetta P. (2006). Human cancer from environmental pollutants: The epidemiological evidence. Mutat. Res. Genet. Toxicol. Environ. Mutagenesis.

[8] Naville D., Gaillard G., Julien B., Vega N., Pinteur C., Chanon S., Vidal H., Le Magueresse-Battistoni B. (2019). Chronic exposure to a pollutant mixture at low doses led to tissue-specific metabolic alterations in male mice fed standard and high-fat high-sucrose diet. Chemosphere.

[9] Fossi M.C. (1998). Biomarkers as diagnostic and prognostic tools for wildlife risk assessment: Integrating endocrine-disrupting chemicals. Toxicol. Ind. Health.

[10] Giesy J.P., Feyk L.A., Jones P.D., Kannan K., Sanderson T. (2003). Review of the effects of endocrine-disrupting chemicals in birds. Pure Appl. Chem..

[11] Stoker T.E., Parks L.G., Gray L.E., Cooper R.L. (2000). Endocrine-disrupting chemicals: Prepubertal exposures and effects on sexual maturation and thyroid function in the male rat. A focus on the EDSTAC recommendations. Crit. Rev. Toxicol..

[12] Zheng X., Zhang K., Zhao Y., Fent K. (2021). Environmental chemicals affect circadian rhythms: An underexplored effect influencing health and fitness in animals and humans. Environ. Int..

[13] Jatkowska N., Kudłak B., Lewandowska P., Liu W., Williams M.J., Schiöth H.B. (2021). Identification of synergistic and antagonistic actions of environmental pollutants: Bisphenols A, S and F in the presence of DEP, DBP, BADGE and BADGE 2HCl in three component mixtures. Sci. Total Environ..

[14] Singh N., Gupta V.K., Kumar A., Sharma B. (2017). Synergistic effects of heavy metals and pesticides in living systems. Front. Chem..

[15] Carnevali O., Santangeli S., Forner-Piquer I., Basili D., Maradonna F. (2018). Endocrine-Disrupting Chemicals in Aquatic Environment: What Are the Risks for Fish Gametes?. Fish Physiol. Biochem..

[16] Kudłak B., Jatkowska N., Liu W., Williams M.J., Barcelo D., Schiöth H.B. (2022). Enhanced Toxicity of Bisphenols Together with UV Filters in Water: Identification of Synergy and Antagonism in Three-Component Mixtures. Molecules.

[17] Lee D.-H., David R.J. (2015). Methodological issues in human studies of endocrine disrupting chemicals. Rev. Endocr. Metab. Disord..

[18] Zhang S., Kang Q., Peng H., Ding M., Zhao F., Zhou Y., Dong Z., Zhang H., Yang M., Tao S. (2019). Relationship between perfluorooctanoate and perfluorooctane sulfonate blood concentrations in the general population and routine drinking water exposure. Environ. Int..

[19] Liu W., Cao H., Liao S., Kudłak B., Williams M.J., Schiöth H.B. (2021). Dibutyl phthalate disrupts conserved circadian rhythm in Drosophila and human cells. Sci. Total Environ..

[20] Brunner M., Tobias S. (2006). Transcriptional and post-transcriptional regulation of the circadian clock of cyanobacteria and Neurospora. Genes Dev..

[21] Gachon F., Nagoshi E., Brown S.A., Ripperger J., Schibler U. (2004). The mammalian circadian timing system: From gene expression to physiology. Chromosoma.

[22] Partch C.L., Carla B.G., Joseph S.T. (2014). Molecular architecture of the mammalian circadian clock. Trends Cell Biol..

[23] Patke A., Michael W.Y., Sofia A. (2020). Molecular mechanisms and physiological importance of circadian rhythms. Nat. Rev. Mol. Cell Biol..

[24] Refinetti R., Michael M. (1992). The circadian rhythm of body temperature. Physiol. Behav..

[25] Guido M.E., Monjes N.M., Wagner P.M., Salvador G.A. (2022). Circadian Regulation and Clock-Controlled Mechanisms of Glycerophospholipid Metabolism from Neuronal Cells and Tissues to Fibroblasts. Mol. Neurobiol..

[26] Challet E. (2019). The circadian regulation of food intake. Nat. Rev. Endocrinol..

[27] Taniguchi H., Fernández A.F., Setién F., Ropero S., Ballestar E., Villanueva A., Yamamoto H., Imai K., Shinomura Y., Esteller M. (2009). Epigenetic Inactivation of the Circadian Clock Gene BMAL1 in Hematologic Malignancies BMAL1 Epigenetic Inactivation. Cancer Res..

[28] Miller B.H., McDearmon E.L., Panda S., Hayes K.R., Zhang J., Andrews J.L., Antoch M.P., Walker J.R., Esser K.A., Hogenesch J.B. (2007). Circadian and CLOCK-controlled regulation of the mouse transcriptome and cell proliferation. Proc. Natl. Acad. Sci. USA.

[29] Storch K.-F., Lipan O., Leykin I., Viswanathan N., Davis F.C., Wong W.H., Weitz C.J. (2002). Extensive and divergent circadian gene expression in liver and heart. Nature.

[30] Gauger M.A., Aziz S. (2005). Cryptochrome, circadian cycle, cell cycle checkpoints, and cancer. Cancer Res..

[31] Savvidis C., Michael K. (2012). Circadian rhythm disruption in cancer biology. Mol. Med..

[32] Kelleher F.C., Aparna R., Anne M. (2014). Circadian molecular clocks and cancer. Cancer Lett..

[33] Gaddameedhi S., Selby C.P., Kaufmann W.K., Smart R.C., Sancar A. (2011). Control of skin cancer by the circadian rhythm. Proc. Natl. Acad. Sci. USA.

[34] Jiang W., Zhao S., Jiang X., Zhang E., Hu G., Hu B., Zheng P., Xiao J., Lu Z., Lu Y. (2016). The circadian clock gene Bmal1 acts as a potential anti-oncogene in pancreatic cancer by activating the p53 tumor suppressor pathway. Cancer Lett..

[35] Lagunas-Rangel F.A., Kudłak B., Liu W., Williams M.J., Schiöth H.B. (2021). The potential interaction of environmental pollutants and circadian rhythm regulations that may cause leukemia. Crit. Rev. Environ. Sci. Technol..

[36] Lagunas-Rangel F.A., Wen L., Helgi B. (2022). Can Exposure to Environmental Pollutants Be Associated with Less Effective Chemotherapy in Cancer Patients?. Int. J. Environ. Res. Public Health.

[37] Lagunas-Rangel F.A., Linnea-Niemi J.V., Kudłak B., Williams M.J., Jönsson J., Schiöth H.B. (2022). Role of the synergistic interactions of environmental pollutants in the development of cancer. GeoHealth..

[38] Lukashina N., Williams M.J., Kartysheva E., Virko E., Kudłak B., Fredriksson R., Spjuth O., Schiöth H.B. (2021). Integrating statistical and machine-learning approach for meta-analysis of bisphenol A-exposure datasets reveals effects on mouse gene expression within pathways of apoptosis and cell survival. Int. J. Mol. Sci..

[39] Vollmers C., Satchidananda P., Luciano D. (2008). A high-throughput assay for siRNA-based circadian screens in human U2OS cells. PLoS ONE.

[40] Hirota T., Lee J.W., Lewis W.G., Zhang E.E., Breton G., Liu X., Garcia M., Peters E.C., Etchegaray J.-P., Traver D. (2010). High-throughput chemical screen identifies a novel potent modulator of cellular circadian rhythms and reveals CKIα as a clock regulatory kinase. PLoS Biol..

[41] Hoffmann J., Symul L., Shostak A., Fischer T., Naef F., Brunner M. (2014). Non-circadian expression masking clock-driven weak transcription rhythms in U2OS cells. PLoS ONE.

[42] Williams M.J., Cao H., Lindkvist T., Mothes T.J., Schiöth H.B. (2020). Exposure to the Environmental Pollutant Bisphenol A Diglycidyl Ether (BADGE) Causes Cell over-Proliferation in Drosophila. Environ. Sci. Pollut. Res. Int..

[43] Williams M.J., Wang Y., Klockars A., Monica Lind P., Fredriksson R., Schiöth H.B. (2014). Exposure to bisphenol A affects lipid metabolism in Drosophila melanogaster. Basic Clin. Pharmacol. Toxicol..

[44] Cao H. (2018). Exposure to xenobiotic chemicals disrupts metabolism, rhythmicity and cell proliferation in Drosophila melanogaster. Ph.D. Thesis.

[45] Ramanathan C., Khan S.K., Kathale N.D., Xu H., Liu A.C. (2012). Monitoring cell-autonomous circadian clock rhythms of gene expression using luciferase bioluminescence reporters. JoVE J. Vis. Exp..

[46] Isojima Y., Nakajima M., Ukai H., Fujishima H., Yamada R.G., Masumoto K., Kiuchi R., Ishida M., Ukai-Tadenuma M., Minami Y. (2009). CKI/-dependent phosphorylation is a temperature-insensitive, period-determining process in the mammalian circadian clock. Proc. Natl. Acad. Sci. USA.

[47] Maier B., Wendt S., Vanselow J.T., Wallach T., Reischl S., Oehmke S., Schlosser A., Kramer A. (2009). A large-scale functional RNAi screen reveals a role for CK2 in the mammalian circadian clock. Genes Dev..

[48] Wood A.J., Lo T.-W., Zeitler B., Pickle C.S., Ralston E.J., Lee A.H., Amora R., Miller J.C., Leung E., Meng X. (2011). Targeted genome editing across species using ZFNs and TALENs. Science.

[49] Gupta R.M., Kiran M. (2014). Expanding the genetic editing tool kit: ZFNs, TALENs, and CRISPR-Cas9. J. Clin. Investig..

[50] Makarova K.S., Haft D.H., Barrangou R., Brouns S.J.J., Charpentier E., Horvath P., Moineau S., Mojica F.J.M., Wolf Y.I., Yakunin A.F. (2011). Evolution and classification of the CRISPR–Cas systems. Nat. Rev. Microbiol..

[51] Hsu P.D., Eric S.L., Feng Z. (2014). Development and applications of CRISPR-Cas9 for genome engineering. Cell.

[52] Doudna J.A., Emmanuelle C. (2014). The new frontier of genome engineering with CRISPR-Cas9. Science.

[53] Nishimasu H., Shi X., Ishiguro S., Gao L., Hirano S., Okazaki S., Noda T., Abudayyeh O.O., Gootenberg J.S., Mori H. (2018). Engineered CRISPR-Cas9 nuclease with expanded targeting space. Science.

[54] Gong K.W., Zhao W., Li N., Barajas B., Kleinman M., Sioutas C., Horvath S., Lusis A.J., Nel A., Araujo J.A. (2007). Air-pollutant chemicals and oxidized lipids exhibit genome-wide synergistic effects on endothelial cells. Genome Biol..

[55] Palanivel R., Vinayachandran V., Biswal S., Deiuliis J.A., Padmanabhan R., Park B., Gangwar R.S., Durieux J.C., Ebreo Cara E.A., Das L. (2020). Exposure to air pollution disrupts circadian rhythm through alterations in chromatin dynamics. Iscience.

[56] Benedusi M., Frigato E., Bertolucci C., Valacchi G. (2021). Circadian deregulation as possible new player in pollution-induced tissue damage. Atmosphere.

