# Supplementary material for: Generation of Endogenous Promoter-Driven Luciferase Reporter System Using CRISPR/Cas9 for Investigating Transcriptional Regulation of the Core Clock Gene BMAL1
Source: Biomedicines. 2022 Dec 1;10(12):3108. doi: 10.3390/biomedicines10123108 (PMC9775583; doi:10.3390/biomedicines10123108)
Supplement: Supplementary file 1 [file biomedicines-10-03108-s001.zip › biomedicines-2021266-supplementary.pdf]

Supplementary Material

```
  1 ggtggaggtc caagtttgtg cctggaactc aagaggaggc tacagctacc
 51 tagacatagg ctttctccca tgtggggcat aatctcttca gagagagaag
101 gctaatgaag gttcagagat aatgcctaga agggtgatta gtaagcatat
151 cagaaggtat taaaagtcat taagtgaaaa gagtttgctt ttcccatcac
201 atccatcttt ttctcccctg ggctttctag atctgttttt ttcctaaact
251 ctcccttcct atttgccttt ctttttgtaa ctcctcttaa ctggttacat
301 cagattatta aaataagctt gactcaaata tttttaacat ttactaaatt
351 ttaagcgctt tgtacttatt tctaaaatga tcatttgatt ccttagagct
401 gtctcttcac aacgtaaccc cacttatttc ttacgtattt ttaaaaaatt
451 ggttctttcc tctaaaaaac agcgatataa aaaaagatag ccatgcagcc
501 caaaaagcag catctcaccc taccattaaa tgtaactttc ttattaaaaa
551 tgtgcttagg aattgcttac ttaatctgaa gatgctttaa aaaagaaatc
601 actgaccagt ctttatctcc tcccacaggt gagaaccccc acataggtat
651 agacatgatt gacaacgacc aaggatcaag tagtcccagt aatgatgagg
701 cagcaatggc tgtcatcatg agcctcttgg aagcagatgc tggactgggt
```

751 ggccctgttg actttagtga cttgccatgg ccgctg~~taa~~*g caacaaactt*

801 ctctctgctg aaacaagccg gagatgtcga agagaatcct ggaccg

Supplementary Material S1. Left homologous arm was designed 786 nt upstream the terminal code site of *BMAL1*. The homologous sequence of P2A-luciferase-EGFP-CRE-SV40-puromycin expression cassette was underline. Terminal code of BMAL1 TAA was removed from the oligo DNA. The *italic* was a P2A sequence coding 2A self-cleaving peptide to separate polypeptides form one expression cassette.

1 acactacatg ttgctttggc aacagctata gtatcaaagt gcattactgg
51 tggagtttta cagtctgtga agcttactgg ataaggagag aatagctttt
101 atgtactgac ttcataaaag ccatctcaga gccattgata caagtcaatc
151 ttactatatg taacttcaga caaagtggaa ctaagcctgc tccagtgttt
201 cctcatcatt gattattggg ctagctgtgg atagcttgca ttaattgtat
251 attttggatt ctgtttgtgt tgaatttttt aatcattgtg cacagaagca
301 tcattggtag cttttatatg caaatggtca tttcagatgt atggtgtttt
351 tacactacaa agaagtcccc catgtggata tttcttatac taattgtatc
401 ataaagccgt ttattcttcc ttgtaagaat cctttactat aaatatgggt
451 taaagtataa tgtactagac agttaaatat ttttaataaa tgtttccctt
501 gttctataaa tactgttcac atttcaaata attagaaaaa aaatcctacg
551 tgttgcaggg ctggattata agtttctaca gtgggtgttc aaggatgatc
601 tacgccagta gcatttgtgg tgcttttctg gccttcataa cgattctggg
651 cttgcataat ttccccatgc tactagctta gctgtgttgt taaaatagtt
701 catttataaa ttgttgcaat tttaatttat gtgagaacaa agatgttact
751 gcttatccac aaacatcgaa tcctctgaaa ttataaattc aagattatat
801 ttaacaaatt ggcttctcta atgctgcacc tctct

Supplementary Material S2. Right homologous arm was designed 835 nt downstream the terminal code site of *BMAL1*. The homologous sequence of P2A-luciferase-EGFP-CRE-SV40-puromycin expression cassette was underline.

tggaagacgccaaaaacataaagaaaggcccggcgccattctatcctctagaggatggaaccgctggagagcaactgcataaggct
atgaagagatacgccctggttcctggaacaattgcttttacagatgcacatatcgaggtgaacatcacgtacgcggaatacttcgaaatg
tccgttcggttggcagaagctatgaaacgatatgggctgaatacaaatcacagaatcgtcgtatgcagtgaaaactctcttcaattctttat
gccggtgttgggcgcgttatttatcggagttgcagttgcgcccgcgaacgacatttataatgaacgtgaattgctcaacagtatgaacatt
tcgcagcctaccgtagtgtttgtttccaaaaaggggttgcaaaaaattttgaacgtgcaaaaaaaattaccaataatccagaaaattattat
catggattctaaaacggattaccagggatttcagtcgatgtacacgttcgtcacatctcatctacctcccggttttaatgaatacgattttgta
ccagagtcctttgatcgtgacaaaacaattgcactgataatgaattcctctggatctactgggttacctaagggtgtggcccttccgcata
gaactgcctgcgtcagattctcgcatgccagagatcctatttttggcaatcaaatcattccggatactgcgattttaagtgttgttccattcca
tcacggttttggaatgtttactacactcggatatttgatatgtggatttcgagtcgtcttaatgtatagatttgaagaagagctgtttttacgatc
ccttcaggattacaaaattcaaagtgcgttgctagtaccaaccctattttcattcttcgccaaaagcactctgattgacaaatacgatttatct
aatttacacgaaattgcttctgggggcgcacctctttcgaaagaagtcggggaagcggttgcaaaacgcttccatcttccagggatacg
acaaggatatgggctcactgagactacatcagctattctgattacacccgagggggatgataaaccgggcgcggtcggtaaagttgtt
ccattttttgaagcgaaggttgtggatctggataccgggaaaacgctgggcgttaatcagagaggcgaattatgtgtcagaggacctat
gattatgtccggttatgtaaacaatccggaagcgaccaacgccttgattgacaaggatggatggctacattctggagacatagcttactg
ggacgaagacgaacacttcttcatagttgaccgcttgaagtctttaattaaatacaaaggatatcaggtggcccccgctgaattggaatc
gatattgttacaacaccccaacatcttcgacgcgggcgtggcaggtcttcccgacgatgacgccggtgaacttcccgccgccgttgttg
ttttggagcacggaaagacgatgacggaaaaagagatcgtggattacgtcgccagtcaagtaacaaccgcgaaaaagttgcgcgga
ggagttgtgtttgtggacgaagtaccgaaaggtcttaccggaaaactcgacgcaagaaaaatcagagagatcctcataaaggccaag
aagggcggaaagtccaaattggctagcggatccggaatggtgagcaagggcgaggagctgttcaccggggtggtgcccatcctggt
cgagctggacggcgacgtaaacggccacaagttcagcgtgtccggcgagggcgagggcgatgccacctacggcaagctgaccct
gaagttcatctgcaccaccggcaagctgcccgtgccctggcccaccctcgtgaccaccctgacctacggcgtgcagtgcttcagccg
ctaccccgaccacatgaagcagcacgacttcttcaagtccgccatgcccgaaggctacgtccaggagcgcaccatcttcttcaaggac
gacggcaactacaagacccgcgccgaggtgaagttcgagggcgacaccctggtgaaccgcatcgagctgaagggcatcgacttca
aggaggacggcaacatcctggggcacaagctggagtacaactacaacagccacaacgtctatatcatggccgacaagcagaagaa
cggcatcaaggtgaacttcaagatccgccacaacatcgaggacggcagcgtgcagctcgccgaccactaccagcagaacacccccc
atcggcgacggccccgtgctgctgcccgacaaccactacctgagcacccagtccgccctgagcaaagaccccaacgagaagcgc
gatcacatggtcctgctggagttcgtgaccgccgccgggatcactctcggcatggacgagctgtacaagtccggactcagatctcgag
ctcaagcttcgaattctgcatccaatttactgaccgtacaccaaaatttgcctgcattaccggtcgatgcaacgagtgatgaggttcgcaa
gaacctgatggacatgttcagggatcgccaggcgttttctgagcatacctggaaaatgcttctgtccgtttgccggtcgtgggcggcatg
gtgcaagttgaataaccggaaatggtttcccgcagaacctgaagatgttcgcgattatcttctatatcttcaggcgcgcggtctggcagta
aaaactatccagcaacatttgggccagctaaacatgcttcatcgtcggtccgggctgccacgaccaagtgacagcaatgctgtttcact
ggttatgcggcggatccgaaaagaaaacgttgatgccggtgaacgtgcaaaacaggctctagcgttcgaacgcactgatttcgacca
ggttcgttcactcatggaaaatagcgatcgctgccaggatatacgtaatctggcatttctggggattgcttataacaccctgttacgtatag
ccgaaattgccaggatcagggttaaagatatctcacgtactgacggtgggagaatgttaatccatattggcagaacgaaaacgctggtt
agcaccgcaggtgtagagaaggcacttagcctgggggtaactaaactggtcgagcgatggatttccgtctctggtgtagctgatgatc
cgaataactacctgttttgccgggtcagaaaaaatggtgttgccgcgccatctgccaccagccagctatcaactcgcgccctggaagg
gatttttgaagcaactcatcgattgatttacggcgctaaggatgactctggtcagagatacctggcctggtctggacacagtgcccgtgt
cggagccgcgcgagatatggcccgcgctggagtttcaataccggagatcatgcaagctggtggctggaccaatgtaaatattgtcatg
aactatatccgtaacctggatagtgaaacaggggcaatggtgcgcctgctggaagatggcgattaagctagcgttaactctagagatcc
gcggccgcatcgattcgaccctgtggaatgtgtgtcagttagggtgtggaaagtccccaggctccccagcaggcagaagtatgcaaa
gcatgcatctcaattagtcagcaaccaggtgtggaaagtccccaggctccccagcaggcagaagtatgcaaagcatgcatctcaatta
gtcagcaaccatagtcccgcccctaactccgcccatcccgcccctaactccgcccagttccgcccattctccgccccatggctgactaa
ttttttttatttatgcagaggccgaggccgcctcggcctctgagctattccagaagtagtgaggaggcttttttggaggcctaggcttttgc
aaaaagcttaccatgaccgagtacaagcccacggtgcgcctcgccacccgcgacgacgtccccagggccgtacgcaccctcgccg
ccgcgttcgccgactaccccgccacgcgccacaccgtcgatccggaccgccacatcgagcgggtcaccgagctgcaagaactcttc
ctcacgcgcgtcgggctcgacatcggcaaggtgtgggtcgcggacgacggcgccgcggtggcggtctggaccacgccggagag

cgtcgaagcgggggcggtgttcgccgagatcggcccgcgcatggccgagttgagcggttcccggctggccgcgcagcaacagat
ggaaggcctcctggcgccgcaccggcccaaggagcccgcgtggttcctggccaccgtcggcgtctcgcccgaccaccagggcaa
gggtctgggcagcgccgtcgtgctccccggagtggaggcggccgagcgcgccggggtgcccgccttcctggagacctccgcgcc
ccgcaacctccccttctacgagcggctcggcttcaccgtcaccgccgacgtcgaggtgcccgaaggaccgcgcacctggtgcatga
cccgcaagcccggtgcctgac

Supplementary Material S3. The DNA sequence of P2A-EGFP-CRE-Puro cassette from addgene (#67503). It is located from 7752-12193 on pF CAG luc-EGFP-cre puro (https://www.addgene.org/browse/sequence/120118/).
